# Landscape Perception Identification and Classification Based on Electroencephalogram (EEG) Features

**DOI:** 10.3390/ijerph19020629

**Published:** 2022-01-06

**Authors:** Yuting Wang, Shujian Wang, Ming Xu

**Affiliations:** 1 Henan Key Laboratory of Earth System Observation and Modeling, Henan University, Kaifeng 475004, China; wyt2019@vip.henu.edu.cn (Y.W.); wangsj@henu.edu.cn (S.W.); 2 College of Geography and Environmental Science, Henan University, Kaifeng 475004, China; 3 BNU-HKUST Laboratory for Green Innovation, Advanced Institute of Natural Sciences, Beijing Normal University at Zhuhai, Zhuhai 519087, China

**Keywords:** unmanned aerial vehicle (UAV), electroencephalogram (EEG) features, landscape perception, machine learning

## Abstract

This paper puts forward a new method of landscape recognition and evaluation by using aerial video and EEG technology. In this study, seven typical landscape types (forest, wetland, grassland, desert, water, farmland, and city) were selected. Different electroencephalogram (EEG) signals were generated through different inner experiences and feelings felt by people watching video stimuli of the different landscape types. The electroencephalogram (EEG) features were extracted to obtain the mean amplitude spectrum (MAS), power spectrum density (PSD), differential entropy (DE), differential asymmetry (DASM), rational asymmetry (RASM), and differential caudality (DCAU) in the five frequency bands of delta, theta, alpha, beta, and gamma. According to electroencephalogram (EEG) features, four classifiers including the back propagation (BP) neural network, k-nearest neighbor classification (KNN), random forest (RF), and support vector machine (SVM) were used to classify the landscape types. The results showed that the support vector machine (SVM) classifier and the random forest (RF) classifier had the highest accuracy of landscape recognition, which reached 98.24% and 96.72%, respectively. Among the six classification features selected, the classification accuracy of MAS, PSD, and DE with frequency domain features were higher than those of the spatial domain features of DASM, RASM and DCAU. In different wave bands, the average classification accuracy of all subjects was 98.24% in the gamma band, 94.62% in the beta band, and 97.29% in the total band. This study identifies and classifies landscape perception based on multi-channel EEG signals, which provides a new idea and method for the quantification of human perception.

## 1. Introduction

Traditionally, landscape classification has always been concerned with descriptive analysis involving the physical characteristics of a landscape based on basic surveys and specifications. However, the combination of the subjective, visual appreciation of scenery and the more objectively describable physical elements seems to have been strong resistance to the idea of landscape classification [1,2]. Yet there seems to be no classification problems if we restrict ourselves to the more limited physical (namely landform and land-use) concept of landscape. Recent studies have evaluated the consistency of the existing manually constructed natural landscape classification with a machine learning-based approach in order to explain the variable importance of the differentiation between natural landscape types [3]. Landscape classification of Central Europe was based on the cluster analysis of principal components, which would be used for further assessment of ecosystem services within the focus region [4]. The later 3S technology also provides a strong driving force for landscape classification research. The idea of using GEOBIA and a supervised classifier to classify ground features and extract landscape information from UAV images has been recognized by most researchers [5]. At present, multi temporal remote sensing images, which have been widely applied, can effectively improve the accuracy of feature information extraction because they can provide spectral information on different time phases [6,7,8]. In the field of remote sensing, various depth learning technologies and high-resolution images have made indelible contributions to landscape classification. However, these are classified based on the objective feature attributes of the landscape itself, and there are relatively few studies on objective measurement and recognition based on landscape perception.

The traditional research methods of landscape perception mainly focus on scenic beauty evaluation (SBE) [9,10,11], the semantic differential method (SD) [12,13], and the analytic hierarchy process (AHP) [14]. The landscape is displayed through photos or field observation. For example, the aesthetic appearance of photographic images are used as a monitoring tool for coral reefs [15]. In addition, subjective perception judgments have been used on different types of pictures to quantify 47 sites along the Colorado River in the Grand Canyon National Park [16]. Qualitative and semi-quantitative research is conducted on the landscape by the fuzzy evaluation method of landscape description and overall perception [17]. However, from the perspective of psychology, Smith believes that the color of the plant itself can help us relieve stress [18]. The human eyes are particularly sensitive to green. The body will automatically reduce excitement when it sees green. At the same time, the “savanna hypothesis”, the “refuge theory” [19], and the “biological theory” [20] all show that there is a genetic basis for the human preference for landscape. Evolutionary psychology also shows that people’s pro naturalness is associated with basic survival needs, such as seeking refuge [15,21,22]. From the perspective of human perception, these notions suggest that people will produce different physiological responses in different environments or landscapes, which are related to the unique pattern of brain activity. Therefore, based on physiological electroencephalography (EEG), we can provide a new view of landscape analysis. The above shows that people will have different physiological reactions when they see different landscapes or when they are in different environments.

The measurement of brain activity is an objective way of assessing the physiological perception of engagement with the landscape, environment, or other objects [23,24,25]. Brain imaging is helpful to measure the effects of unconscious stimuli [26,27]. EEG frequency features have commonly been used in EEG signals. Generally, the frequency range of high amplitude signals observed is different when subjects are in calm state and when they are in a stimulated state [28,29]. EEG features (frequency domain features, time domain features, and spatial domain features) in EEG signals represent the brain region activities. With the continuous development of neuroscience and the trend of interdisciplinarity, thus far, there have been many studies using EEG technology and machine learning for recognition with good classification accuracy, e.g., emotion recognition [30,31,32,33], object structure recognition [34,35], color recognition [36], landscape and animal image recognition [37]. Some studies have gradually applied EEG technology to different fields, including environmental perception and landscape assessment [38,39,40,41], while others have also explored the impact of specific environmental characteristics on the natural environment [42,43,44]. Therefore, this study combines the experimental technology of neurology and UAV aerial video to identify and classify the landscape types from the perspective of human perception and compares the advantages and disadvantages of different classification features and classification methods.

## 2. Materials and Methods

### 2.1. Materials

**Figure 1:**
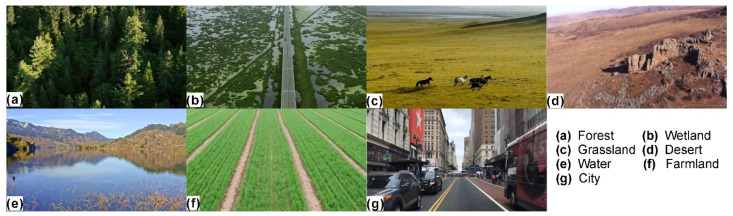
Experimental video materials.

Five landscape videos were selected for each landscape type. Seven professors with more than five years of experience in landscape design were asked to rate each landscape video on a scale of 1–10. The highest-scoring landscape videos were chosen to become the experimental materials according to the cumulative scores. The stimuli included seven high-definition videos, mainly including different landscape types (forest, wetland, grassland, desert, water, farmland, and city) (Figure 1). The subjects were stimulated through video experience. During the experiment, conductive paste was used to reduce scalp resistance below 5 KΩ. In the laboratory, the room was completely closed and without noise. A 15-inch display device was put in front of the subjects. There were seven different videos, each of which remained on screen for about 40 s (2 repeats for each stimuli video). After a video was displayed, the volunteers had a minute to rest, mainly to calm their emotions and to not affect the next video. Then the next landscape video was played, followed by a rest, and so on until the end of the whole experiment. The whole process lasted for about 25 min. The sequence in which the videos played during the experiment is random.

### 2.2. Subjects

A total of 20 volunteers aged between 25 and 55, 12 women and 8 men, participated in the experimental procedure. The participants had varied employment situations, such as university staff, social workers of various industries, and university graduates. We described the purpose of the experiment before beginning, and participants were asked to be right-handed, with no color blindness, and in good physical health, without any history of a mental disorder. Additionally, a video was played in the lab, during which the participants were asked to stay as still as possible so that interferences such as EMG did not increase. If they met the requirements and agreed to continue the experiment, they then signed an informed consent form before testing. The study was approved by the school’s ethics committee.

### 2.3. Method

**Figure 2:**
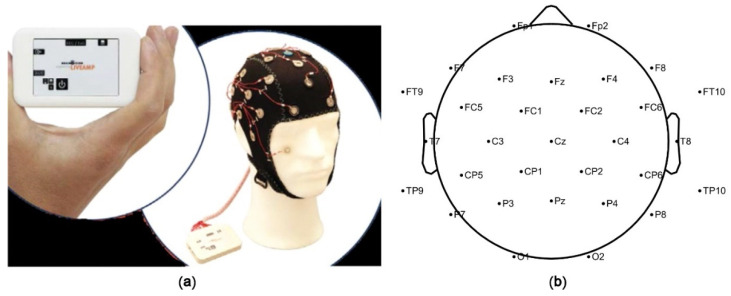
Brain Products (LiveAmp) in the experiment. (**a**) Brain Products (LiveAmp) with 32 channels; (**b**) The electrode position of international 10–20 system with 32 channels.

In this study, the Active System produced by Brain Products (LiveAmp) with 32 channels was used to obtain the signals of the brain activity (Figure 2). The experiment used the international 10–20 system and a 32-channel electrode cap. The video stimuli were displayed in random order, and each landscape type was repeated twice for a total of 14 short videos. The original EEG data were analyzed by the EEGlab, which is a toolbox for processing continuous EEG signals.

### 2.4. Statistical Analysis

In this study, frequency domain features and spatial domain features are selected as important indicators of landscape recognition. The frequency domain features include: mean amplitude spectrum (MAS) [45], power spectrum density (PSD), and Differential Entropy (DE) [33,46,47], and spatial domain features include: differential asymmetry (DASM) and rational asymmetry (RASM) [48], and differential caudality (DCAU) [33].

The EEG signals were detrended using the average of left and right mastoids as a reference. Then, 250 Hz downsampling and 0.5–70 Hz filtering were performed to obtain the preprocessed EEG datasets. Third, the eye electrical, electromyography, electrocardiography, power frequency interference, and other disturbance artefacts were removed by independent component analysis (ICA) [49,50,51,52]. Next, the EEG signals were segmented into contiguous 2 s windows, and any segments which retained artefacts were rejected [53,54]. Therefore, a total of 5600 (7 × 2 × 20 × 20) data samples were generated. In other words, there were a total of 7 landscape categories, and each video stimulus was repeated twice. Each subject could get 20 segments for 2 s of each video stimulus, with a total of 20 subjects. Then, Fast Fourier transform was used to extract frequency band information. The frequency-domain features were extracted to obtain the logarithmic frequency energy values of the waves in five frequency bands: delta (1–4 Hz), theta (4–8 Hz), alpha (8–13 Hz), beta (13–30 Hz), and gamma (30–70 Hz) [55]. The sample size of each subject in each frequency band is 280 characteristic data. Each frequency band contains the data of 29 electrode channels, and the total frequency band contains 145 electrode channels, that is, the samples of 5 frequency bands have a structure of 29 × 280, the sample structure of the total frequency band is 145 × 280 (Table 1).

Firstly, four classifiers including the back propagation (BP) neural network algorithm (BP), k-nearest neighbor classification algorithm (KNN), random forest algorithm (RF), and support vector machine algorithm (SVM) were used to classify different landscape types of brain waves after the pretreatment of the EEG signals. Then, the recognition accuracy in different situations was obtained, and the accuracy of different classification methods for landscape type recognition was compared, in order to reflect the differences in people’s perceptions of different landscape types.

The EEG data were randomly divided into training (80%) and test (20%) data. For our purposes, we have used 10-fold cross-validation to train and test extracted features for all classifiers. For KNN, we used k = 5 for a baseline in comparison with other classifiers. For random forest, we constructed the tree number with 500. We used LIBSVM software [56] to implement the SVM classifier and employ linear kernel, the back propagation (BP) neural network algorithm was also used. 

**Figure 3:**
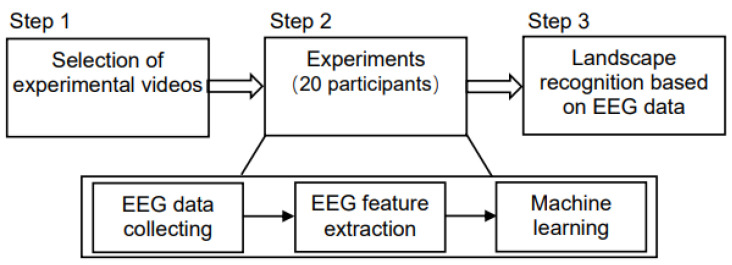
Flow chart of the experiment.

Statistical analysis and data processing were mainly completed using MATLAB and R language. Figure 3 displays the experimental process.

## 3. Results

### 3.1. The Classification Effect of Different Classifiers

**Figure 4:**
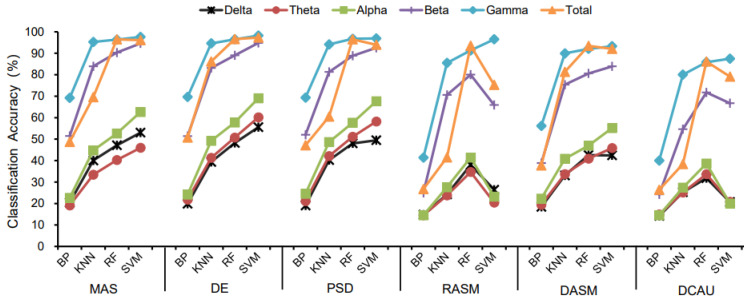
The classification results of different classifiers.

**Figure 5:**
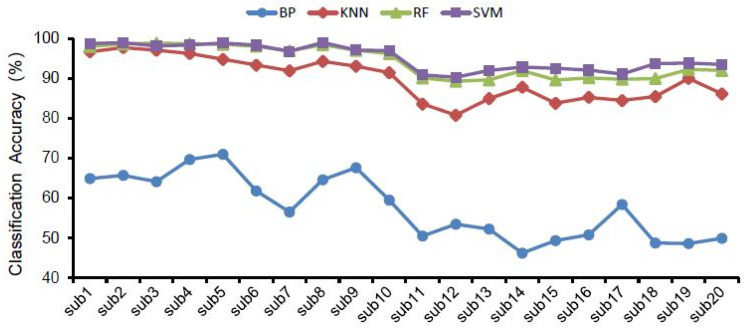
The highest classification accuracy of different classifiers in all bands in 20 subjects.

The classification accuracy of the four classifiers for different landscape types are displayed in Table 2 and Figure 4. For all EEG features, the SVM classifier and RF classifier had the highest landscape recognition accuracy, followed by KNN, and BP had the lowest classification accuracy among the four classifiers. Meanwhile, Figure 5 shows the highest classification accuracy of different classifiers in 20 groups of subjects in all bands of brain waves, and also shows that the classification accuracy of SVM and RF was higher than that of KNN and BP.

Specifically, in all wave bands of delta, theta, alpha, beta, and gamma and EEG features including ES, PSD and DE, the SVM classifier had the highest classification accuracy, followed by the RF classifier. In the total band of EEG features such as MAS and PSD, the RF classifier had a slightly higher classification accuracy than the SVM classifier, then the accuracy of the SVM classifier and the RF classifier was significantly higher than the KNN classifier and the BP classifier.

Among the delta, theta, alpha, and beta wave bands of EEG features including RASM and DCAU, the RF classifier had the best classification effect, followed by the SVM classifier. Only in the gamma band, the SVM classifier had a higher classification accuracy than the RF classifier, the KNN classifier, and the BP classifier.

In the delta and total waves of EEG features DASM, the classification accuracy of the RF classifier was higher than that of the SVM classifier, while in the theta, alpha, beta, and gamma bands, the classification accuracy of the SVM classifier was higher than that of the KNN classifier and the BP classifier.

Using the different classifiers, the trend of landscape recognition accuracy was SVM and RF > KNN > BP, while similar trends were confirmed using both the goodness of fit and sensitivity (Table S1).

### 3.2. The Classification Effect of Different EEG Features

**Figure 6:**
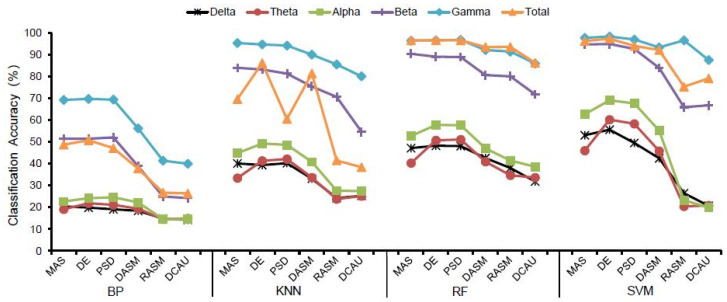
Classification accuracy of different EEG features.

**Figure 7:**
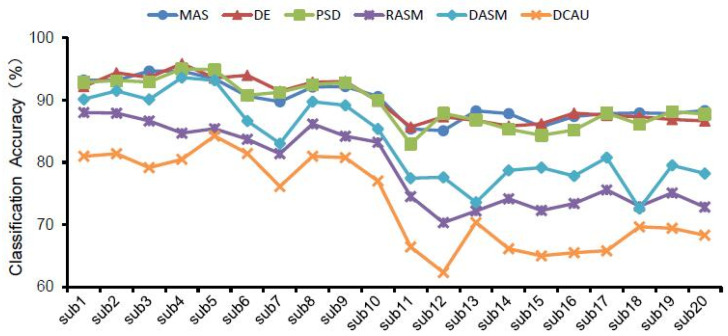
The highest classification accuracy of different EEG features in all bands in 20 subjects.

In general, for the same brain waves and the same classifier (Table 2 and Figure 6), the classification accuracy of frequency domain features including MAS, PSD and DE was higher than that of spatial domain features including DASM, RASM and DCAU indicators among the six EEG features selected. Furthermore, Figure 7 shows the maximum classification accuracy of different EEG features in 20 subjects in different waves, and also demonstrates that the classification accuracy of frequency domain features is higher than that of spatial domain features.

For the BP classifier, the DE feature had the highest classification accuracy in the delta, theta, gamma, and total wave bands, then PSD was the highest in the alpha band and MAS was the highest in the beta band.

For the KNN classifier, the highest classification accuracy was the PSD feature in the delta and theta wave bands, the DE feature had the highest accuracy in the alpha and total wave bands, and the MAS feature had the highest accuracy in the beta and gamma bands.

For the RF classifier, the classification accuracy of the PSD feature was the highest in the delta, theta, alpha and gamma wave bands, the classification accuracy of the MAS feature was the highest in the beta band, and the accuracy of the DE feature was the highest in the total band.

### 3.3. The Classification Effect of Different Brainwave Bands

With the same selection of classifier and EEG features (Figure 4 and Figure 6), the classification accuracy was highest in the gamma, beta and total bands, and was relatively lower in the delta, theta and alpha bands. Therefore, the recognition of landscape perception by high-frequency waves was more effective than that of low-frequency waves. In all wave bands, the mean classification accuracy of all subjects was 98.24% in the gamma band, 97.29% in the total band, and 94.83% in the beta band. The classification accuracy of the delta band, theta band and alpha band was 55.56%, 60.17% and 69.04%, respectively. This indicates that the classification accuracy of high-frequency waves was higher than that of low-frequency waves in landscape perception recognition and classification. The difference in classification accuracy of different subjects may be related to the subjects’ landscape preference (Figure 5 and Figure 7).

In the BP, KNN and SVM classifiers, the classification accuracy of the gamma band and beta band was higher than that of the total band based on EEG features such as MAS and PSD. The classification accuracy of the gamma band was higher than that of the total band based on EEG features including DE, DASM, RASM, and DCAU. In the RF classifier, there was little difference between the classification accuracy of the gamma band and the total band.

## 4. Discussion

The power of EEG signals in the frequency domain is one of the most commonly used EEG features for emotion analysis. Generally, when the subject is in a calm state, the high amplitude signals in the low frequency range will be more obvious, while in a stimulus state, the high amplitude signals in the high frequency range will be more obvious [28,29]. EEG features (e.g., frequency domain features, features, and spatial domain features) in an EEG signal represent the activities in each brain region. At present, research on recognition and classification based on EEG technology and machine learning have achieved good results.

This study showed that the SVM classifier has the highest classification accuracy in this study. This has been well established in many application fields of EEG technology. In emotion recognition analysis, the frequency domain features that power the spectral density (PSD) of the EEG signals and the asymmetry indexes of the 12 electrode pairs were used to classify discrete emotional states (disgust, happiness, neutrality, sadness, and tenseness) induced by watching a video. A previous study obtained results on the classification accuracy of within-stimulus and found 93.3% accuracy in the PSD feature, and 85.4% accuracy in asymmetry features [30]. Shahabi et al. distinguished emotional states (happy, neutral, and melancholy) based on EEG features, and concluded that the classification accuracy between happy and neutral was 93.7%, while the classification accuracy between happy and melancholy was 80.4% using the SVM classifier [32]. Rasheed et al. classified the EEG signals as red, green, and blue colors and successfully classified the three visual conditions having accuracies of 84%, 89% and 98% with linear, polynomial, and radial basis function kernels, respectively, within all the groups of data among all the subjects [36]. The extracted PSD features were classified using two-level SVM classifiers in corresponding object-shape classes (including cone, cube, cylinder, sphere, prism, hemisphere, pyramid (square base), hexagonal base cylinder, lock, and mouse) for the three experimental phases. The recognition accuracy was 88.34% for pure touch and 81.1% for pure vision, the recognition accuracy of the mixture of tactile and auditory was 82.2%, and the average classification accuracy of the three target recognition modes was 83.89% [34]. Based on the EEG of prefrontal brain area, SVM was used as a classifier to detect driver fatigue, and the accuracy was 85% [57]. The recognition of three human stress levels was characterized based on the relative difference between beta and alpha in EEG signals using SVM as classifier, with a recognition accuracy of 75% [58].

The results show that DE features had the highest classification accuracy in all waves including delta, theta, alpha, beta and gamma and total waves for the SVM classifier. Therefore, DE is an important indicator of landscape perception classification based on EEG technology. Similarly, in the experiment on emotion recognition, using DE as a feature proxy achieves higher recognition accuracy than other features. Zheng et.al used differential entropy, which is a measure of the amount of information included in EEG signals, as the input of deep belief networks, they also used SVM and KNN for the classification of emotional states (positive, neutral, and negative). The classification accuracy was respectively 86.08%, 83.99%, and 72.60% [33]. Differential entropy was also employed to distinguish the positive, neutral, and negative emotional states induced by videos, where the CNN received input as the classifier. The classification accuracy was 83.8% [31].

This study also showed that the classification accuracy of high-frequency waves was higher than that of low-frequency waves in landscape perception recognition and classification. Moreover, there was similar research suggesting that the gamma band (roughly 30–100 Hz) was suitable for EEG-based emotion classification [59]. The similarity with the classification of people’s emotions shows that different landscapes do cause subtle changes in people’s emotions. At the same time, it also proves that it is feasible to identify people’s perception of different landscapes through multi-dimensional EEG features [60]. This result is of great significance for evaluating the impact of landscapes on people, which also has important reference value for the quantification of ecosystem cultural services [44,61].

When the total band of 145 dimensions was used for landscape perception recognition, it did not show the highest accuracy, as it was even lower than the accuracy of landscape perception recognition in the beta band and gamma band. Therefore, more dimensions were not always better [59,62]. In this study, the highest classification accuracy of a landscape video was 98.24% in the gamma band using the SVM classifier. Rus et al. also confirmed that the EEG features of the gamma band are more suitable for object recognition. The classification accuracy of three different classifiers (SVM, KNN and ANN) was 89.5%, 89.5% and 83%, respectively [35], which is consistent with the conclusion that the classification accuracy of the gamma band is the highest of all bands in this study. There are relatively few studies on the recognition of landscape types based on EEG technology. Although Lam et al. implemented the single-layer neural network to recognize and classify landscape and animal pictures based on EEG data, the average accuracy was 91.15%, in which the average recognition accuracy of landscape pictures was 89.69% and that of animal pictures was 92.34% [37]. However, Lam’s research only identified landscape pictures and animal pictures, and did not further identify and classify landscape type pictures.

Compared with the traditional recognition research based on the attribute characteristics of the landscape, this research identifies and classifies landscape perception based on the characteristics of UAV aerial video and EEG features, which provides a certain reference value for the objective quantification of landscape perception evaluation in the future. At the same time, it also provides new ideas and methods for landscape evaluation research, which is worthy of more comprehensive and in-depth research. Future research should focus on the differences in individual perception of landscape physiology, including an increase in sample size and age range and the change of working conditions, as well as further differences in gender, education level, residence, property income and other social attributes. Further research also needs to expand the scope of landscape types. At the same time, more experimental case studies are also necessary. It is very necessary to confirm the conclusions of this study in more complex situations and make them more robust. This study also provides a reference for cultural services of ecosystem services. Compared with the traditional landscape perception evaluation of landscape ecosystems based on questionnaire surveys, more objective multi-dimensional EEG features may be used as a reference in the future.

With the application of brain computer interface technology becoming more and more developed and market-oriented, it is believed that there will be many successful case studies in landscape evaluation and tourism evaluation in the future. There is a need for further research on the relationship between brain activity and landscapes with different characteristics since it is still in the exploratory stage, and there are few relevant studies and generally accepted theoretical basis to explain it. Therefore, it is still a great challenge to verify these relationships with more extensive experimental cases.

## 5. Conclusions

This study identifies and classifies landscape types based on multi-channel and multi-dimensional EEG features, explores algorithms suitable for landscape perception recognition and classification, preliminarily defines the most suitable frequency band for landscape perception recognition and classification, and compares EEG features with better effects of landscape perception knowledge. It provides a certain reference for landscape perception evaluation in the future.

The results confirmed the following points. For all EEG features, the SVM and RF classifiers have the highest classification accuracy. When the bands are the same and the classifiers are the same, the classification accuracy of frequency domain features, MAS, PSD and DE are relatively higher than those of spatial domain features DASM, RASM and DCAU. The DE feature is the most effective among all of the classifiers. For the same classifier and the same EEG feature, the classification accuracy of the beta band and the gamma band is the highest, while the classification accuracy of the delta, theta and alpha bands is relatively lower. Therefore, the high frequency signals are more effective in landscape perception recognition.

## Figures and Tables

**Table 1:** Dimensions of each EEG feature.

Feature	Delta	Theta	Alpha	Beta	Gamma	Total
MAS	29	29	29	29	29	145
PSD	29	29	29	29	29	145
DE	29	29	29	29	29	145
DASM	13	13	13	13	13	65
RASM	13	13	13	13	13	65
DCAU	11	11	11	11	11	55

**Table 2:** The accuracy of landscape perception and recognition based on different EEG features and different classifiers.

Feature	Classifier	Delta (%)	Theta (%)	Alpha (%)	Beta (%)	Gamma (%)	Total (%)
MAS	BP	20.38 ± 5.74	19.08 ± 5.82	22.67 ± 7.18	51.45 ± 11.49	69.2 ± 11.15	48.69 ± 10.25
	KNN	40.07 ± 11.85	33.39 ± 11.2	44.79 ± 15.1	83.91 ± 11.98	95.28 ± 4.17	69.54 ± 18.81
	RF	47.13 ± 10.54	40.27 ± 12.56	52.63 ± 14.69	90.33 ± 6.77	96.36 ± 3.32	96.51 ± 3.33
	SVM	53.02 ± 11.51	45.96 ± 12.74	62.62 ± 15.82	94.62 ± 4.95	97.63 ± 2.82	96.13 ± 4.38
PSD	BP	19.08 ± 5.41	21.09 ± 6.09	24.55 ± 8.11	52.01 ± 12.16	69.36 ± 11.39	47.12 ± 12.28
	KNN	40.21 ± 10.63	42.1 ± 12.61	48.63 ± 17.1	81.28 ± 14.66	94.13 ± 5.48	60.47 ± 18.52
	RF	48.02 ± 11.85	51.07 ± 14.58	57.64 ± 16.24	88.84 ± 8.12	96.72 ± 3.10	96.52 ± 3.34
	SVM	49.47 ± 8.95	58.23 ± 12.38	67.62 ± 14.36	92.55 ± 6.63	96.90 ± 3.13	93.98 ± 6.79
DE	BP	19.86 ± 5.93	21.84 ± 6.79	24.14 ± 8.27	51.41 ± 12.22	69.65 ± 11.51	50.71 ± 9.21
	KNN	39.36 ± 11.32	41.29 ± 15.24	49.2 ± 17.41	83.13 ± 12.83	94.62 ± 4.71	86.14 ± 11.32
	RF	48.21 ± 10.84	50.71 ± 13.91	57.73 ± 15.94	89.03 ± 8.67	96.51 ± 3.15	96.61 ± 3.33
	SVM	55.56 ± 9.72	60.17 ± 14.13	69.04 ± 14.76	94.83 ± 4.7	98.24 ± 2.31	97.29 ± 3.09
DASM	BP	18.41 ± 4.83	19.39 ± 6.12	22.24 ± 7.46	38.82 ± 11.99	56.13 ± 13.26	37.74 ± 10.63
	KNN	33.09 ± 8.71	33.64 ± 13.03	40.80 ± 18.19	75.36 ± 14.15	89.93 ± 8.53	81.32 ± 11.64
	RF	42.57 ± 8.2	40.87 ± 12.86	47.01 ± 16.3	80.59 ± 9.46	92.08 ± 5.86	93.45 ± 5.52
	SVM	42.44 ± 8.12	45.82 ± 12.67	55.14 ± 15.99	83.88 ± 9.49	93.26 ± 6.29	92.08 ± 7.04
RASM	BP	14.83 ± 3.78	14.54 ± 3.29	14.54 ± 3.87	24.93 ± 10.84	41.38 ± 14.35	26.71 ± 10.20
	KNN	24.23 ± 6.90	23.78 ± 8.03	27.62 ± 10.12	70.59 ± 13.21	85.46 ± 8.79	41.49 ± 13.01
	RF	37.99 ± 8.42	34.66 ± 10.43	41.42 ± 14.73	80.02 ± 11.95	91.35 ± 6.59	93.49 ± 5.70
	SVM	26.42 ± 6.63	20.43 ± 6.72	23.19 ± 10.37	65.88 ± 14.20	96.52 ± 3.35	75.27 ± 12.52
DCAU	BP	14.22 ± 2.72	14.88 ± 3.16	14.54 ± 2.99	24.23 ± 9.06	39.96 ± 13.42	26.37 ± 10.53
	KNN	25.25 ± 9.00	25.13 ± 8.56	27.39 ± 8.04	54.62 ± 16.93	80.01 ± 8.11	38.36 ± 18.20
	RF	31.81 ± 11.14	33.64 ± 11.57	38.60 ± 10.90	71.76 ± 13.07	85.86 ± 9.42	86.16 ± 10.63
	SVM	20.71 ± 6.28	20.81 ± 7.49	19.96 ± 6.81	66.71 ± 10.82	87.46 ± 7.29	79.09 ± 8.97

Notes: Mean ± SD.

## Data Availability

The data presented in this study are available in insert article and Supplementary Materials.

## Supplementary Materials

The following are available online at https://www.mdpi.com/article/10.3390/ijerph19020629/s1, Table S1: The goodness of fit, specificity, and sensitivity of different classifiers in different landscape types.
