# Supplementary material for: Landscape Perception Identification and Classification Based on Electroencephalogram (EEG) Features
Source: Int J Environ Res Public Health. 2022 Jan 6;19(2):629. doi: 10.3390/ijerph19020629 (PMC8776197; doi:10.3390/ijerph19020629)
Supplement: Supplementary file 1 [file ijerph-19-00629-s001.zip › ijerph-1501122-supplementary.pdf]

Table S1. The goodness of fit, specificity and sensitivity of different classifiers in different landscape types.

		MAS			PSD			DE			DASM			RASM			DCAU		
**Classifier**	**Landscape**	**Goodness of fit**	**Sensitivity**	**Specificity**	**Goodness of fit**	**Sensitivity**	**Specificity**	**Goodness of fit**	**Sensitivity**	**Specificity**	**Goodness of fit**	**Sensitivity**	**Specificity**	**Goodness of fit**	**Sensitivity**	**Specificity**	**Goodness of fit**	**Sensitivity**	**Specificity**
Delta		0.22			0.18			0.23			0.17			0.02			0.00		
BP	Wetland		0.13	0.96		0.10	0.97		0.16	0.95		0.07	0.98		0.02	0.98		0.02	0.98
BP	Grassland		0.18	0.90		0.10	0.93		0.18	0.89		0.14	0.92		0.03	0.97		0.01	0.99
BP	Water		0.30	0.78		0.31	0.79		0.28	0.78		0.29	0.77		0.11	0.85		0.15	0.86
BP	Forest		0.34	0.73		0.40	0.67		0.31	0.76		0.36	0.71		0.54	0.41		0.68	0.34
BP	Desert		0.23	0.82		0.23	0.81		0.25	0.84		0.23	0.81		0.19	0.83		0.12	0.88
BP	Cropland		0.11	0.92		0.09	0.93		0.15	0.90		0.12	0.91		0.03	0.97		0.02	0.98
BP	City		0.28	0.98		0.22	0.98		0.33	0.96		0.25	0.97		0.04	0.98		0.04	0.98
Theta		0.27			0.29			0.29			0.22			0.01			0.00		
BP	Wetland		0.13	0.96		0.11	0.97		0.18	0.96		0.10	0.97		0.02	0.98		0.03	0.99
BP	Grassland		0.21	0.90		0.17	0.92		0.25	0.89		0.18	0.89		0.03	0.97		0.03	0.98
BP	Water		0.31	0.78		0.29	0.79		0.28	0.80		0.30	0.78		0.13	0.85		0.13	0.89
BP	Forest		0.34	0.76		0.41	0.71		0.36	0.78		0.29	0.76		0.62	0.37		0.74	0.30
BP	Desert		0.28	0.83		0.26	0.83		0.28	0.83		0.29	0.82		0.13	0.87		0.12	0.89
BP	Cropland		0.16	0.91		0.18	0.92		0.18	0.90		0.15	0.91		0.02	0.98		0.02	0.98
BP	City		0.29	0.97		0.29	0.97		0.31	0.97		0.28	0.97		0.04	0.98		0.01	0.99
Alpha		0.32			0.35			0.35			0.28			0.00			0.00		
BP	Wetland		0.20	0.96		0.19	0.97		0.23	0.95		0.14	0.97		0.02	0.98		0.03	0.98
BP	Grassland		0.24	0.90		0.26	0.91		0.24	0.90		0.22	0.89		0.02	0.98		0.03	0.98
BP	Water		0.31	0.80		0.31	0.82		0.28	0.82		0.29	0.81		0.16	0.87		0.11	0.90
BP	Forest		0.31	0.78		0.38	0.76		0.34	0.77		0.34	0.76		0.64	0.41		0.73	0.31
BP	Desert		0.28	0.82		0.31	0.82		0.25	0.83		0.29	0.82		0.18	0.83		0.11	0.88
BP	Cropland		0.18	0.91		0.20	0.92		0.18	0.91		0.19	0.91		0.03	0.97		0.01	0.98
BP	City		0.34	0.97		0.34	0.97		0.35	0.97		0.29	0.97		0.05	0.97		0.04	0.98
Beta		0.77			0.76			0.76			0.64			0.36			0.36		
BP	Wetland		0.59	0.97		0.62	0.97		0.61	0.97		0.44	0.98		0.18	0.97		0.22	0.97
BP	Grassland		0.55	0.92		0.56	0.92		0.51	0.92		0.41	0.89		0.22	0.91		0.20	0.92
BP	Water		0.53	0.89		0.50	0.89		0.50	0.90		0.41	0.86		0.32	0.81		0.34	0.82
BP	Forest		0.51	0.90		0.52	0.89		0.52	0.88		0.38	0.85		0.44	0.73		0.42	0.73
BP	Desert		0.54	0.90		0.53	0.90		0.51	0.89		0.44	0.85		0.33	0.84		0.27	0.79
BP	Cropland		0.50	0.92		0.44	0.93		0.46	0.92		0.33	0.92		0.24	0.93		0.16	0.92
BP	City		0.62	0.97		0.67	0.97		0.59	0.97		0.52	0.97		0.29	0.97		0.23	0.98
Gamma		0.88			0.85			0.88			0.82			0.64			0.64		
BP	Wetland		0.76	0.99		0.71	0.98		0.79	0.98		0.69	0.98		0.42	0.97		0.44	0.97
BP	Grassland		0.74	0.94		0.67	0.94		0.70	0.95		0.56	0.94		0.45	0.91		0.44	0.91
BP	Water		0.72	0.94		0.69	0.93		0.76	0.94		0.61	0.91		0.46	0.86		0.50	0.87
BP	Forest		0.69	0.94		0.68	0.92		0.70	0.94		0.60	0.90		0.45	0.85		0.48	0.85
BP	Desert		0.76	0.93		0.70	0.92		0.74	0.94		0.62	0.91		0.47	0.88		0.45	0.87

BP	Cropland		0.66	0.96		0.59	0.95		0.67	0.95		0.57	0.94		0.46	0.93		0.36	0.93
BP	City		0.77	0.98		0.76	0.98		0.74	0.98		0.68	0.97		0.52	0.97		0.54	0.97
Total		0.77			0.71			0.81			0.67			0.39			0.39		
BP	Wetland		0.59	0.97		0.57	0.95		0.64	0.97		0.52	0.96		0.31	0.95		0.30	0.94
BP	Grassland		0.48	0.92		0.46	0.92		0.50	0.91		0.38	0.89		0.29	0.90		0.24	0.91
BP	Water		0.47	0.90		0.45	0.89		0.47	0.91		0.41	0.88		0.34	0.84		0.40	0.84
BP	Forest		0.48	0.88		0.47	0.88		0.48	0.90		0.41	0.85		0.35	0.83		0.44	0.80
BP	Desert		0.47	0.88		0.47	0.88		0.53	0.89		0.38	0.87		0.32	0.84		0.32	0.84
BP	Cropland		0.43	0.92		0.45	0.92		0.46	0.92		0.36	0.91		0.28	0.90		0.26	0.92
BP	City		0.61	0.97		0.63	0.96		0.65	0.97		0.53	0.96		0.36	0.95		0.37	0.96
Delta		0.18			0.17			0.19			0.15			0.03			0.06		
KNN	Wetland		0.39	0.90		0.39	0.90		0.42	0.90		0.34	0.89		0.23	0.85		0.33	0.85
KNN	Grassland		0.32	0.89		0.27	0.88		0.31	0.89		0.29	0.87		0.19	0.89		0.20	0.89
KNN	Water		0.32	0.90		0.34	0.89		0.32	0.88		0.26	0.87		0.29	0.84		0.25	0.83
KNN	Forest		0.37	0.88		0.31	0.87		0.30	0.87		0.32	0.86		0.18	0.89		0.18	0.86
KNN	Desert		0.43	0.91		0.40	0.91		0.38	0.91		0.24	0.90		0.18	0.90		0.24	0.89
KNN	Cropland		0.40	0.88		0.38	0.89		0.37	0.90		0.33	0.89		0.24	0.88		0.17	0.89
KNN	City		0.66	0.96		0.68	0.95		0.67	0.96		0.57	0.95		0.43	0.88		0.41	0.92
Theta		0.19			0.21			0.20			0.17			0.03			0.02		
KNN	Wetland		0.41	0.88		0.45	0.90		0.42	0.89		0.35	0.87		0.16	0.90		0.17	0.90
KNN	Grassland		0.33	0.89		0.32	0.89		0.34	0.89		0.36	0.86		0.22	0.87		0.25	0.86
KNN	Water		0.32	0.90		0.31	0.91		0.34	0.90		0.30	0.90		0.24	0.85		0.19	0.88
KNN	Forest		0.35	0.89		0.36	0.88		0.39	0.88		0.30	0.89		0.21	0.87		0.25	0.86
KNN	Desert		0.47	0.93		0.48	0.92		0.44	0.92		0.31	0.91		0.37	0.83		0.33	0.86
KNN	Cropland		0.43	0.87		0.40	0.89		0.41	0.89		0.40	0.89		0.21	0.89		0.29	0.85
KNN	City		0.61	0.96		0.62	0.95		0.60	0.95		0.55	0.94		0.28	0.90		0.27	0.91
Alpha		0.22			0.25			0.23			0.19			0.05			0.05		
KNN	Wetland		0.50	0.90		0.50	0.92		0.51	0.91		0.39	0.89		0.23	0.89		0.28	0.88
KNN	Grassland		0.43	0.90		0.42	0.90		0.40	0.90		0.42	0.90		0.29	0.88		0.26	0.86
KNN	Water		0.36	0.91		0.37	0.91		0.38	0.91		0.35	0.91		0.25	0.88		0.22	0.90
KNN	Forest		0.45	0.90		0.49	0.89		0.47	0.89		0.38	0.87		0.31	0.83		0.31	0.86
KNN	Desert		0.41	0.92		0.41	0.92		0.40	0.91		0.34	0.90		0.32	0.86		0.30	0.86
KNN	Cropland		0.44	0.90		0.47	0.90		0.46	0.90		0.44	0.89		0.25	0.89		0.28	0.87
KNN	City		0.67	0.95		0.67	0.96		0.67	0.96		0.57	0.95		0.26	0.92		0.27	0.92
Beta		0.76			0.71			0.75			0.69			0.57			0.43		
KNN	Wetland		0.82	0.97		0.82	0.97		0.82	0.97		0.80	0.98		0.79	0.95		0.64	0.94
KNN	Grassland		0.80	0.96		0.75	0.96		0.80	0.95		0.74	0.95		0.76	0.94		0.50	0.92
KNN	Water		0.81	0.97		0.75	0.96		0.77	0.96		0.75	0.95		0.59	0.94		0.50	0.92
KNN	Forest		0.73	0.96		0.71	0.96		0.74	0.96		0.65	0.93		0.66	0.93		0.57	0.93
KNN	Desert		0.78	0.97		0.78	0.95		0.77	0.96		0.67	0.96		0.64	0.94		0.59	0.92
KNN	Cropland		0.80	0.96		0.75	0.95		0.78	0.96		0.71	0.96		0.58	0.96		0.48	0.92
KNN	City		0.93	0.99		0.91	0.99		0.91	0.99		0.92	0.99		0.84	0.98		0.81	0.97
Gamma		0.91			0.91			0.91			0.90			0.81			0.69		
KNN	Wetland		0.95	0.99		0.94	0.99		0.95	0.99		0.92	0.99		0.90	0.99		0.78	0.97

KNN	Grassland		0.95	0.99		0.96	0.99		0.95	0.98		0.93	0.98		0.88	0.98		0.73	0.95
KNN	Water		0.93	0.99		0.93	0.98		0.95	0.98		0.91	0.98		0.84	0.96		0.73	0.94
KNN	Forest		0.91	0.99		0.92	0.99		0.89	0.99		0.88	0.98		0.88	0.97		0.76	0.97
KNN	Desert		0.92	0.99		0.91	0.99		0.90	0.99		0.90	0.98		0.87	0.98		0.77	0.95
KNN	Cropland		0.95	0.99		0.92	0.99		0.94	0.99		0.92	0.99		0.86	0.98		0.76	0.97
KNN	City		0.97	1.00		0.97	0.99		0.96	1.00		0.95	1.00		0.90	0.99		0.91	0.99
Total		0.55			0.40			0.77			0.77			0.16			0.13		
KNN	Wetland		0.70	0.96		0.61	0.94		0.87	0.98		0.87	0.98		0.38	0.93		0.44	0.87
KNN	Grassland		0.68	0.93		0.56	0.91		0.81	0.96		0.77	0.96		0.46	0.87		0.31	0.86
KNN	Water		0.66	0.95		0.50	0.93		0.83	0.96		0.80	0.96		0.37	0.92		0.29	0.93
KNN	Forest		0.66	0.93		0.56	0.91		0.79	0.97		0.76	0.94		0.48	0.81		0.46	0.84
KNN	Desert		0.65	0.95		0.56	0.94		0.81	0.98		0.73	0.97		0.48	0.88		0.42	0.90
KNN	Cropland		0.68	0.94		0.60	0.92		0.83	0.97		0.80	0.97		0.31	0.92		0.33	0.91
KNN	City		0.87	0.99		0.77	0.97		0.93	0.99		0.92	1.00		0.44	0.97		0.43	0.96
Delta		0.23			0.24			0.23			0.22			0.15			0.10		
RF	Wetland		0.49	0.90		0.51	0.90		0.50	0.90		0.41	0.91		0.40	0.88		0.35	0.89
RF	Grassland		0.34	0.90		0.34	0.91		0.34	0.91		0.34	0.90		0.29	0.89		0.20	0.89
RF	Water		0.41	0.91		0.41	0.89		0.40	0.91		0.38	0.88		0.36	0.89		0.25	0.87
RF	Forest		0.39	0.91		0.38	0.91		0.38	0.90		0.35	0.90		0.26	0.90		0.21	0.88
RF	Desert		0.49	0.91		0.50	0.92		0.50	0.92		0.39	0.91		0.41	0.90		0.35	0.87
RF	Cropland		0.44	0.92		0.44	0.91		0.44	0.91		0.42	0.91		0.32	0.90		0.24	0.88
RF	City		0.78	0.95		0.78	0.94		0.76	0.95		0.73	0.94		0.69	0.93		0.62	0.91
Theta		0.27			0.28			0.30			0.24			0.13			0.09		
RF	Wetland		0.55	0.90		0.51	0.90		0.55	0.90		0.42	0.90		0.34	0.90		0.32	0.89
RF	Grassland		0.38	0.91		0.39	0.92		0.41	0.92		0.38	0.90		0.26	0.91		0.30	0.89
RF	Water		0.40	0.91		0.42	0.92		0.42	0.92		0.37	0.91		0.34	0.89		0.27	0.90
RF	Forest		0.47	0.91		0.46	0.91		0.48	0.90		0.37	0.90		0.36	0.89		0.26	0.89
RF	Desert		0.58	0.92		0.59	0.92		0.58	0.92		0.49	0.91		0.46	0.90		0.42	0.88
RF	Cropland		0.46	0.92		0.46	0.92		0.44	0.92		0.45	0.91		0.31	0.89		0.30	0.88
RF	City		0.70	0.95		0.72	0.95		0.75	0.95		0.68	0.94		0.52	0.90		0.52	0.90
Alpha		0.30			0.34			0.33			0.26			0.18			0.11		
RF	Wetland		0.58	0.92		0.58	0.92		0.60	0.93		0.45	0.90		0.39	0.90		0.37	0.91
RF	Grassland		0.46	0.92		0.46	0.93		0.52	0.92		0.50	0.91		0.44	0.90		0.33	0.89
RF	Water		0.47	0.91		0.49	0.92		0.48	0.93		0.46	0.92		0.39	0.90		0.32	0.89
RF	Forest		0.51	0.92		0.54	0.92		0.53	0.92		0.34	0.90		0.38	0.89		0.40	0.89
RF	Desert		0.48	0.92		0.50	0.92		0.53	0.92		0.44	0.90		0.40	0.89		0.37	0.89
RF	Cropland		0.53	0.92		0.57	0.92		0.57	0.92		0.45	0.91		0.37	0.91		0.33	0.89
RF	City		0.73	0.95		0.75	0.95		0.74	0.96		0.68	0.95		0.56	0.92		0.48	0.91
Beta		0.85			0.84			0.83			0.71			0.69			0.59		
RF	Wetland		0.90	0.98		0.89	0.98		0.90	0.98		0.86	0.96		0.83	0.97		0.71	0.95
RF	Grassland		0.85	0.98		0.82	0.97		0.85	0.98		0.77	0.96		0.78	0.97		0.61	0.94
RF	Water		0.85	0.98		0.85	0.97		0.85	0.97		0.77	0.97		0.75	0.96		0.63	0.95
RF	Forest		0.83	0.97		0.80	0.97		0.81	0.97		0.72	0.95		0.75	0.95		0.66	0.96
RF	Desert		0.83	0.98		0.85	0.97		0.84	0.97		0.74	0.97		0.73	0.96		0.71	0.94

RF	Cropland		0.87	0.98		0.85	0.97		0.85	0.97		0.78	0.96		0.76	0.96		0.71	0.94
RF	City		0.96	0.99		0.98	0.99		0.95	0.99		0.94	0.98		0.92	0.98		0.87	0.97
Gamma		0.94			0.94			0.94			0.91			0.87			0.76		
RF	Wetland		0.97	0.99		0.98	0.99		0.98	0.99		0.96	0.99		0.94	0.99		0.83	0.98
RF	Grassland		0.96	0.99		0.94	0.99		0.96	0.99		0.93	0.99		0.92	0.98		0.83	0.97
RF	Water		0.96	0.99		0.96	0.99		0.94	0.99		0.94	0.99		0.91	0.98		0.81	0.97
RF	Forest		0.96	0.99		0.95	0.99		0.93	0.99		0.89	0.98		0.89	0.98		0.82	0.97
RF	Desert		0.95	1.00		0.97	1.00		0.95	0.99		0.88	0.99		0.89	0.99		0.81	0.96
RF	Cropland		0.96	0.99		0.97	0.99		0.96	0.99		0.93	0.99		0.92	0.99		0.88	0.98
RF	City		0.98	1.00		0.98	1.00		0.97	1.00		0.96	0.99		0.95	1.00		0.92	0.99
Total		0.93			0.94			0.94			0.91			0.91			0.78		
RF	Wetland		0.97	0.99		0.97	0.99		0.97	0.99		0.97	0.99		0.96	0.99		0.84	0.98
RF	Grassland		0.93	0.99		0.96	0.99		0.95	0.99		0.93	0.99		0.91	0.99		0.80	0.97
RF	Water		0.94	0.99		0.95	0.99		0.95	0.99		0.93	0.99		0.94	0.99		0.81	0.96
RF	Forest		0.93	0.99		0.95	0.99		0.94	0.99		0.91	0.99		0.93	0.98		0.83	0.97
RF	Desert		0.95	0.99		0.94	0.99		0.95	0.99		0.91	0.99		0.91	0.99		0.82	0.97
RF	Cropland		0.97	0.99		0.97	1.00		0.96	1.00		0.95	0.99		0.94	0.99		0.87	0.98
RF	City		0.98	1.00		0.99	1.00		0.99	1.00		0.98	1.00		0.98	0.99		0.93	0.99
Delta		0.33			0.29			0.33			0.21			0.04			0.02		
SVM	Wetland		0.54	0.89		0.51	0.88		0.58	0.89		0.40	0.87		0.29	0.85		0.34	0.78
SVM	Grassland		0.46	0.90		0.42	0.89		0.47	0.90		0.38	0.88		0.19	0.87		0.14	0.87
SVM	Water		0.47	0.90		0.49	0.90		0.51	0.91		0.37	0.89		0.29	0.84		0.24	0.83
SVM	Forest		0.41	0.91		0.38	0.91		0.46	0.91		0.37	0.90		0.22	0.89		0.17	0.87
SVM	Desert		0.50	0.93		0.47	0.92		0.50	0.94		0.37	0.92		0.24	0.91		0.15	0.90
SVM	Cropland		0.51	0.93		0.43	0.93		0.50	0.94		0.39	0.92		0.17	0.89		0.14	0.91
SVM	City		0.79	0.98		0.75	0.98		0.79	0.98		0.70	0.96		0.44	0.90		0.27	0.91
Theta		0.41			0.42			0.46			0.28			0.02			0.01		
SVM	Wetland		0.64	0.90		0.62	0.91		0.66	0.91		0.47	0.88		0.23	0.88		0.19	0.85
SVM	Grassland		0.50	0.90		0.52	0.91		0.54	0.92		0.44	0.90		0.21	0.87		0.23	0.82
SVM	Water		0.50	0.92		0.52	0.92		0.56	0.92		0.41	0.90		0.23	0.85		0.12	0.92
SVM	Forest		0.51	0.92		0.56	0.92		0.57	0.93		0.42	0.91		0.25	0.86		0.24	0.81
SVM	Desert		0.60	0.95		0.57	0.95		0.62	0.94		0.50	0.91		0.32	0.87		0.27	0.86
SVM	Cropland		0.60	0.94		0.62	0.95		0.58	0.95		0.45	0.93		0.18	0.87		0.17	0.88
SVM	City		0.76	0.98		0.77	0.98		0.81	0.98		0.69	0.96		0.23	0.91		0.17	0.91
Alpha		0.48			0.50			0.49			0.35			0.04			0.01		
SVM	Wetland		0.70	0.93		0.74	0.93		0.73	0.93		0.58	0.90		0.25	0.86		0.23	0.85
SVM	Grassland		0.60	0.92		0.60	0.92		0.63	0.92		0.58	0.92		0.33	0.86		0.27	0.84
SVM	Water		0.57	0.93		0.60	0.93		0.62	0.93		0.53	0.92		0.27	0.84		0.18	0.87
SVM	Forest		0.60	0.94		0.63	0.93		0.62	0.94		0.46	0.91		0.23	0.86		0.28	0.82
SVM	Desert		0.64	0.95		0.62	0.95		0.59	0.95		0.50	0.93		0.28	0.88		0.26	0.86
SVM	Cropland		0.64	0.95		0.64	0.95		0.62	0.96		0.52	0.93		0.15	0.90		0.13	0.92
SVM	City		0.80	0.98		0.79	0.98		0.82	0.98		0.71	0.97		0.24	0.93		0.13	0.93
Beta		0.93			0.89			0.93			0.75			0.44			0.52		
SVM	Wetland		0.95	0.99		0.93	0.99		0.96	0.99		0.89	0.98		0.75	0.94		0.72	0.94

SVM	Grassland		0.94	0.98		0.90	0.98		0.92	0.99		0.81	0.96		0.68	0.92		0.59	0.93
SVM	Water		0.93	0.99		0.91	0.98		0.95	0.99		0.81	0.96		0.59	0.92		0.61	0.95
SVM	Forest		0.91	0.98		0.89	0.98		0.89	0.98		0.76	0.95		0.61	0.93		0.67	0.94
SVM	Desert		0.93	0.99		0.89	0.98		0.91	0.99		0.75	0.97		0.55	0.94		0.65	0.93
SVM	Cropland		0.93	0.99		0.90	0.99		0.93	0.99		0.79	0.97		0.64	0.95		0.63	0.95
SVM	City		0.98	1.00		0.96	1.00		0.98	1.00		0.92	0.99		0.65	0.97		0.76	0.97
Gamma		0.96			0.95			0.98			0.92			0.76			0.79		
SVM	Wetland		0.99	1.00		0.98	0.99		0.99	1.00		0.96	0.99		0.90	0.97		0.89	0.98
SVM	Grassland		0.97	1.00		0.96	0.99		0.99	1.00		0.94	0.99		0.84	0.96		0.87	0.98
SVM	Water		0.98	0.99		0.97	0.99		0.99	1.00		0.95	0.99		0.83	0.97		0.83	0.97
SVM	Forest		0.96	0.99		0.96	0.99		0.95	1.00		0.90	0.98		0.84	0.97		0.83	0.97
SVM	Desert		0.96	0.99		0.97	0.99		0.97	1.00		0.90	0.99		0.81	0.97		0.83	0.97
SVM	Cropland		0.97	1.00		0.96	1.00		0.99	1.00		0.95	0.99		0.86	0.99		0.87	0.98
SVM	City		0.99	1.00		0.97	1.00		0.99	1.00		0.97	1.00		0.86	0.99		0.91	0.99
Total		0.93			0.90			0.96			0.90			0.61			0.64		
SVM	Wetland		0.96	0.99		0.94	0.99		0.98	1.00		0.96	0.99		0.78	0.96		0.76	0.96
SVM	Grassland		0.94	0.99		0.92	0.98		0.97	0.99		0.91	0.98		0.76	0.95		0.75	0.95
SVM	Water		0.93	0.99		0.93	0.99		0.96	0.99		0.92	0.98		0.75	0.96		0.75	0.95
SVM	Forest		0.94	0.99		0.91	0.99		0.96	0.99		0.87	0.98		0.76	0.95		0.77	0.95
SVM	Desert		0.94	0.99		0.92	0.99		0.96	0.99		0.88	0.98		0.72	0.95		0.72	0.96
SVM	Cropland		0.96	0.99		0.93	0.99		0.98	1.00		0.94	0.99		0.78	0.97		0.73	0.96
SVM	City		0.98	1.00		0.97	1.00		0.98	1.00		0.97	1.00		0.79	0.98		0.83	0.99
